# Urethral caruncle causes acute urinary retention

**DOI:** 10.1002/iju5.12756

**Published:** 2024-07-06

**Authors:** Akifumi Katsu, Masato Yanagi, Norio Motoda, Hiroyoshi Kono, Ryoji Kimata, Tsutomu Hamasaki, Yukihiro Kondo

**Affiliations:** ^1^ Department of Urology Nippon Medical School Musashikosugi Hospital Kawasaki Kanagawa Japan; ^2^ Department of Pathology Nippon Medical School Musashikosugi Hospital Kawasaki Kanagawa Japan; ^3^ Department of Urology Nippon Medical School Hospital Bunkyo‐ku Tokyo Japan

**Keywords:** inspection of urethral meatus, urethral carcinoma, urethral caruncle, urinary retention, women

## Abstract

**Introduction:**

We encountered a case of urinary retention caused by a urethral caruncle.

**Case presentation:**

An 86‐year‐old woman presented to our hospital with urinary retention. When the urinary bladder catheter was placed, a smooth, well‐defined mass 20 mm in diameter was detected on the posterior wall of the external urethral meatus. The patient was diagnosed with urinary retention due to a urethral caruncle, and the mass was resected. The mass was pathologically compatible with a urethral caruncle. The patient could urinate postoperatively. Ultrasound test after surgery showed residual urine volume was 100 mL.

**Conclusion:**

Inspecting the urethral meatus is vital in the clinical examination of older women with voiding symptoms. A urethral caruncle is a rare cause of urinary retention. However, large urethral caruncles are at risk of causing urinary retention suggesting that resecting the urethral caruncles at an appropriate time is desirable.

**Abbreviation & Acronym**
ECOG: Eastern Collaborative Oncology Group

**Keynote message**

A urethral caruncle is a rare cause of urinary retention. However, large urethral caruncles are at risk of causing urinary retention suggesting that resecting the urethral caruncles at an appropriate time is desirable.

## Introduction

The urethral caruncle is defined as a highly vascular polypoid lesion originating from the posterior wall of the external urethral meatus in females and is usually <1 cm.^1^ It is the most common benign urethral tumor in females after menopause.^2^, ^3^ Hypoestrogenemia is the most critical risk factor for its etiology. The urethral caruncle is usually asymptomatic and is incidentally found. However, some patients experience pain, bleeding, or dysuria.^3^ In addition, rare cases of urinary caruncles causing urinary retention have been reported.^1^, ^4^ We encountered a case of urinary retention caused by a urethral caruncle.

## Case presentation

**Fig. 1:**
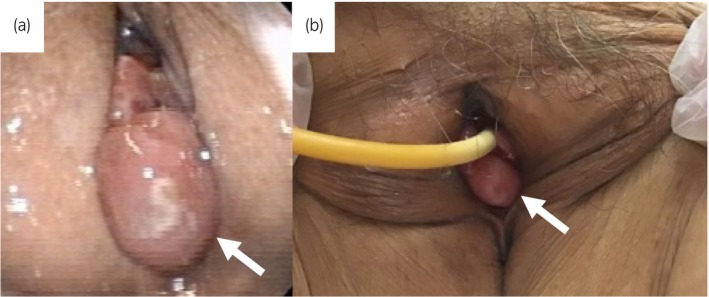
Photographs showing the preoperative appearance of the lesion. (a) Before urinary bladder catheter insertion. The white arrow shows a urethral caruncle. (b) After urinary bladder catheter insertion. The white arrow shows a urethral caruncle.

**Fig. 2:**
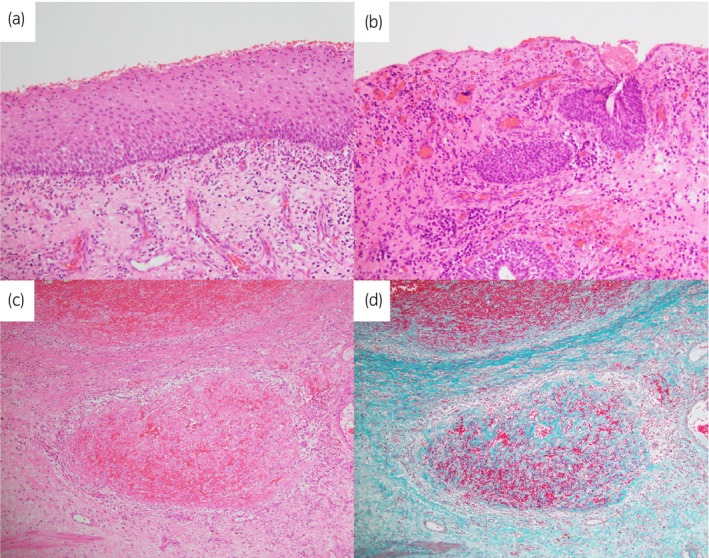
Microscopic image of the tumor. (a) The tumor was covered by keratinized squamous epithelium with neutrophilic infiltration. (b) The tumor partially covered with urothelium with erosion. (c, d) Recanalization of organized thrombus was observed in dilated blood vessels. (d: Elastica Masson‐Goldner staining).

An 86‐year‐old woman with ECOG performance status of 3 presented to our hospital with urinary retention. She had micturition desire. This was his first visit to our hospital. One year ago, she had voiding symptoms, including slow stream and visited a clinic. She was prescribed an anticholinergic agent for 1 month, but she did not visit the clinic after that, probably due to cognitive decline. She had no history of psychiatric diseases or any event such as new medication that would cause urinary retention. Upon inspecting the urethral meatus, a pedunculated mass measuring 20 mm was confirmed on the posterior wall of the external urethral meatus (Fig. 1a). The patient's serum creatinine level was 0.72 mg/dL. A urinary bladder catheter was placed, and 300 mL of urine was drained (Fig. 1b). Cystoscopy showed no abnormalities in the bladder. The patient was diagnosed with urinary retention due to a urethral caruncle and underwent tumor resection under local anesthesia. The huge caruncle was resected with an electric scalpel at the bottom of the caruncle for the purpose of preventing recurrence and hemostasis was achieved with an electric scalpel. Because of the extensive resection surface of the tissue on the external urethral meatus, a urethral catheter was indwelling for 7 days postoperatively to prevent urethral stricture due to wound adhesion. Pathological examination revealed that the tumor was a polypoid lesion covered with non‐neoplastic keratinized squamous epithelium or urothelium with inflammation and dilated blood vessels with thrombus (Fig. 2a,b). These findings were compatible with urethral caruncle (Fig. 2c,d). After removal of the urethral catheter, the patient could urinate. Thought the preoperative and postoperative period, she did not administration of oral cholinergic agonists. She has cognitive decline and is unable to remember the face of her attending physician and her activities of daily living was low. Therefore, pressure‐flow study or uroflowmetry was not performed. However, ultrasound test after surgery showed residual urine volume was about 100 ml. The similar results were obtained in several subsequent post‐discharge outpatient ultrasound test 1, 3, 6, and 12 months after surgery. There was no recurrence of urethral caruncle and urinary retention at least 12 months after surgery.

## Discussion

In the present case, removal of the urethral caruncle made it possible to urinate. We decided neurogenic bladder was negative because the patient had urinary retention with micturition desire and after resection of the urethral caruncle, urination was possible without administration of cholinergic agonists. There was no history of psychiatric illness or any triggering events such as new medications prior to the visit. Unfortunately, there is no preoperative residual urine data because the patient was seen for the first time because of urinary retention. However, based on the above facts, we made a diagnosis of urinary retention due to urethral caruncle.

A urethral caruncle is a rare cause of urinary retention. To our knowledge, only two cases have been previously reported.^1^, ^4^ Table 1 presents the background of the present and previously reported cases. The urethral caruncles in all patients, including the present case, occurred on the posterior wall of the external urethral meatus and were ≥2 cm. These patients underwent resection of the urethral caruncle and could urinate. Enlarged urethral caruncles can lead to urinary retention. The patients had no local symptoms such as bleeding or pain but had long‐term voiding symptoms before urinary retention. These urethral caruncles may gradually increase over time, causing bladder output obstruction, voiding symptoms, and urinary retention. When a patient presents with urinary retention, a urethral caruncle can be easily identified because inspection of the urethral meatus is mandatory. In contrast, urethral caruncles may be overlooked during the clinical examination of patients with voiding symptoms. Furthermore, diseases in the vicinity of the urethra in women, including uterine prolapse, bladder prolapse, and labial adhesions can cause urinary retention. Therefore, inspecting the urethral meatus is vital in examining women, especially older women with voiding symptoms.

**Table 1:** Patient characteristics of previously reported cases and the present case of urinary retention due to urethral caruncle

No.	Year	Author	Age (years)	Size (mm)	Location	Clinical diagnosis	Treatment
1	2014	Coban et al.^1^	41	20	Posterior lip of the urethra	Urethral caruncle	Resection
2	2021	Meutia et al.^4^	83	25	Posterior lip of the urethra	Urethral caruncle	Resection
3	2023	Present case	86	20	Posterior lip of the urethra	Urethral caruncle	Resection

A previous study reported that urethral caruncles are not associated with an increased risk of malignancy.^3^ However, some cases of various neoplasms, including adenocarcinoma, urothelial carcinoma, squamous cell carcinoma, melanoma, lymphoma, and sarcoma, may clinically mimic the urethral caruncle.^5^, ^6^, ^7^, ^8^ Therefore, a pathological diagnosis by biopsy or resection is critical, particularly for large masses.

We have experienced another case of urethral stricture after urethral caruncle surgery. A similar case has also been reported.^9^ Generally, duration of indwelling a urethral catheter after resection of a urethral caruncle is about 2 days. In the present case, duration of indwelling a urethral catheter after the surgery was extended to 7 days, because the resection surface was extensive and the urethra was thought to be tend to urethral stricture. The optimal duration of indwelling a urethral catheter after urethral caruncle resection is controversial, and future studies are required.

## Conclusion

A urethral caruncle is a rare cause of urinary retention. However, large urethral caruncles are at risk of causing urinary retention and are challenging to diagnose clinically, suggesting that resection of urethral caruncles at an appropriate time is desirable.

## Author contributions

Akifumi Katsu: Data curation; writing – original draft. Masato Yanagi: Conceptualization; data curation; writing – original draft. Norio Motoda: Data curation. Hiroyoshi Kono: Data curation. Ryoji Kimata: Data curation. Tsutomu Hamasaki: Conceptualization; data curation; writing – original draft. Yukihiro Kondo: Writing – original draft; writing – review and editing.

## Conflict of interest

The authors declare that they have no competing interests.

## Approval of the research protocol by an Institutional Reviewer Board

Not applicable.

## Informed consent

Informed consent for publication has been obtained from the patient's family described in this case report.

## Registry and the Registration No. of the study/trial

Not applicable.
